# Sacroiliac screws fixation navigated with three-dimensional printing personalized guide template for the treatment of posterior pelvic ring injury: A case report

**DOI:** 10.3389/fsurg.2022.1025650

**Published:** 2023-01-06

**Authors:** Zhanyu Yang, Bin Sheng, Delong Liu, Yiwei Wang, Chao Liu, Rui Xiao

**Affiliations:** ^1^ Department of Orthopedics, Hunan Provincial People's Hospital (the First Affiliated Hospital of Hunan Normal University), Changsha, China; ^2^ Department of Orthopedics, Hunan Emergency Center, Changsha, China

**Keywords:** posterior pelvic ring injury, sacroiliac screw fixation, 3D printing technology, guide template, case report

## Abstract

**Objective:**

Pelvic injuries refer to the disruption of the inherent structural and mechanical integrity of the pelvic ring. Sacroiliac screw fixation technique is often used for the treatment of posterior pelvic ring injury, which is prone to the iatrogenic injury. Various attempts were proposed to avoid iatrogenic injuries, while the executing processes are usually too cumbersome. The patient-personalized guide template based on 3D printing technology has been considered as a promising method, which can achieve lower deviation and higher accuracy in a simple and convenient way. We reported the first case of posterior pelvic ring injury using 3D printing personalized guide template with the verification of intraoperative CT.

**Methods:**

The subject was a 74-year-old female with posterior pelvic ring injury. Two patient-specific guide templates were customized based on 3D printing technology, one for S1 and the other for S2. We used the guide templates for navigation to place the sacroiliac screws. The placement of screws was verified by intraoperative CT. Intraoperative and postoperative variables were collected.

**Results:**

The technique helped us successfully insert the sacroiliac screws into the safe zone. The intraoperative blood loss was 23.03 ml, and the duration of operation was 62 min. The exposure dose during CT scanning was 7.025 mSv. The assessment of screws position was excellent. Furthermore, there was no sign of any functional impairment postoperatively.

**Conclusion:**

Sacroiliac screws fixation with the assistance of 3D printing personalized guide template under the verification of intraoperative CT may be a promising method to treat posterior pelvic ring injuries.

## Introduction

Pelvic injuries refer to the disruption of the inherent structural and mechanical integrity of the pelvic ring, accounting for 3% of all skeletal fractures (1, 2). Despite the lack of bony synostosis, the pelvic ring is highly stable due to the presence of numerous strong and extensive attachments of muscles, tendons, and ligaments (3). Therefore, the pelvic injuries raise more attention since it is most occurred in the setting of high energy and is frequently complicated with additional injuries elsewhere in the body (4). It is reported that the injury of the posterior pelvic ring is significantly associated with the increased morbidity, mainly related to hemorrhagic shock caused by venous plexus injury (5).

With the development and practical application of minimally invasive techniques, percutaneous insertion of cannulated screws into the sacroiliac joint has been applied to the injuries of posterior pelvic ring. Compared to traditional fixation, percutaneous screw fixation technique presents an excellent result, allowing a shorter duration of surgery, a smaller incision, less soft tissue damage and less bleeding (6). There is a safe zone between the neuroforamen and the cortex of the sacrum, which is often wide enough to place sacroiliac screws. However, some patients accompanied with atypical anatomy have narrow corridors so that it is difficult to locate the safe zone during operation. The conventional 2D imaging with mobile C-arm cannot obtain the view of the safe area directly (7). As a result, although the frequency of fluoroscopy exposure is often increased, the incidence of complications cannot be significantly reduced. Under conventional fluoroscopy, the misplacement rate of screws has been reported at 2%–16% (8–10). Sacroiliac screw fixation technique is prone to the iatrogenic complication of vascular and nerve injury, with an incidence of 0.5%–7.7% (11, 12). Because of the limitations of the current percutaneous sacroiliac screw fixation, it is not only the improvement of surgical skills, but also the innovative guidance methods and newly implements have been introduced to increase the accuracy of screws position. Various attempts have been proposed to avoid the misposition of internal plants, such as intraoperative CT, 3D fluoroscopy and computer-assisted techniques (13–16).

These methods may decrease the incidence of iatrogenic injuries and raise the success rate of surgery, while the executing processes are too cumbersome and limited by expensive imaging devices. Recently, a patient-personalized guide template based on 3D printing technology for precise navigation of sacroiliac screws has been considered as a promising method to improve security, which can achieve lower deviation and higher accuracy in a simple and convenient way (17, 18). Moreover, Intraoperative CT can provide vivid and complete three-dimensional imaging and facilitate the location of safe area accurately, which has become a reliable tool for assessing the malposition of internal plants, especially in spine surgery (19).

Here we report a 74-year-old female patient, the first case of posterior pelvic ring injury using 3D printing personalized guide template with the verification of intraoperative CT.

## Case presentation

### Participant

**Figure 1:**
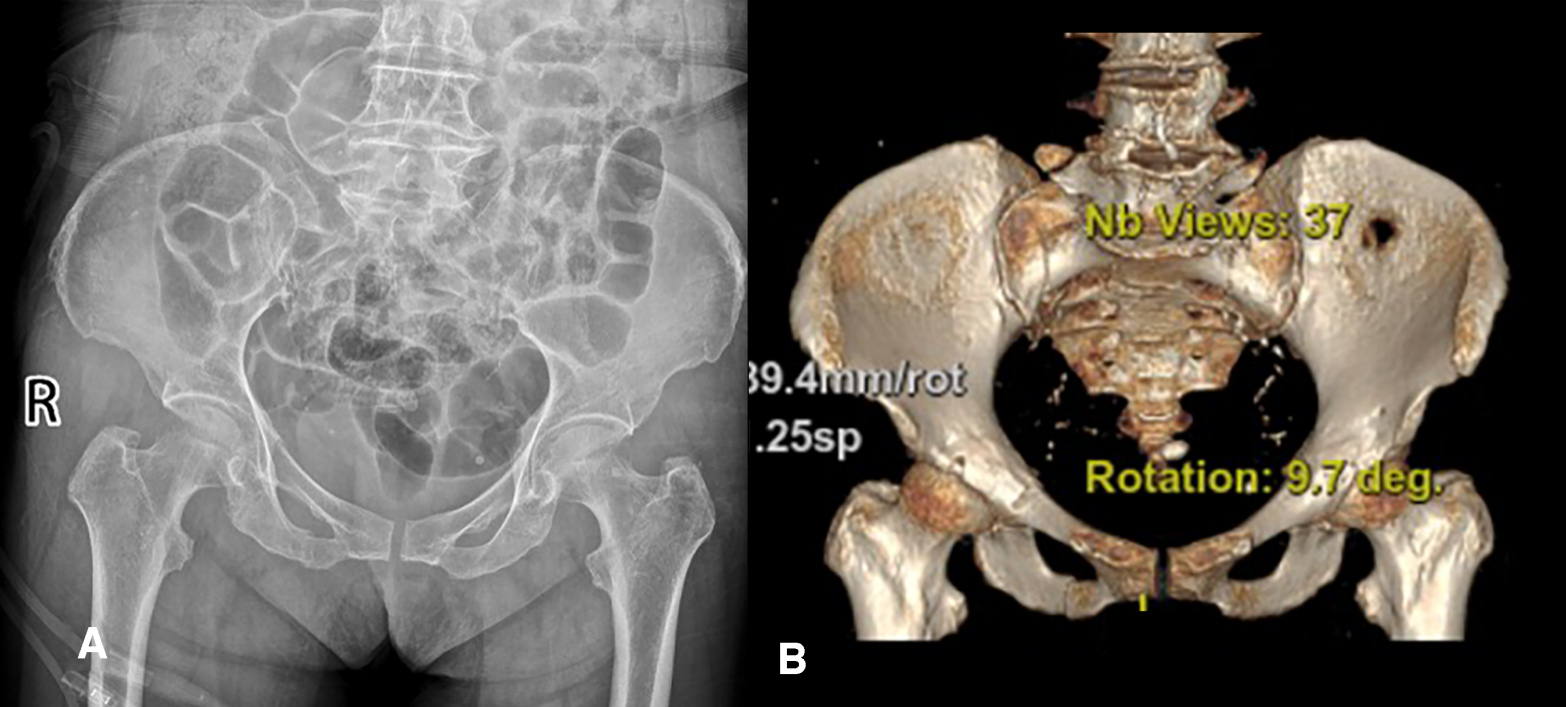
The imaging examination before surgery. (**A**) The anteroposterior radiograph of pelvis. (**B**) Three-dimensional reconstruction of pelvis.

A 74-year-old, previously healthy female patient (weight 62.3 kg, BMI = 25.60 kg/m^2^) was injured in a traffic accident, presented to Department of Orthopedics of our hospital. She sustained a rotationally unstable pelvic posterior ring injury (Tile B2-1) consisting of a right sacroiliac joint fracture, an ipsilateral fracture of upper and lower ramus of pubis and an ipsilateral L5 transverse process fracture (Figure 1). There were no symptoms of sacral nerve injury such as perineum numbness and fecal incontinence. The patient had no previous history of severe trauma and no specific family history. Physical examination revealed the peripheral nervous system was normal, but the involved parts had tenderness and limited hip movement. The laboratory examination demonstrated that there was a mild anemia, and the rest had no obvious abnormality. The imaging examination showed a fracture of upper and lower ramus of right pubis, an ipsilateral sacral crescent fracture and an ipsilateral L5 transverse process fracture.

L5 transverse process fracture indicates the iliolumbar ligament injury, while sacral crescent fracture indicates the interosseous sacroiliac ligament injury. All evidence suggested that this was a partially stable injury of the posterior pelvic ring caused by lateral compression. Given the patient's complaints and examination, percutaneous sacroiliac screws fixation, a minimally invasive technique, was adopted for the treatment. We informed the patient of the potential hazard of such a procedure to blood vessels and nerves. Subsequently, with the consent and request of the patient and his legal guardian, a supplementary plan consisting of percutaneous sacroiliac screws fixation, navigation with three-dimensional printing personalized guide template and verification with intraoperative CT was proposed and accepted.

### Materials preparation

**Figure 2:**
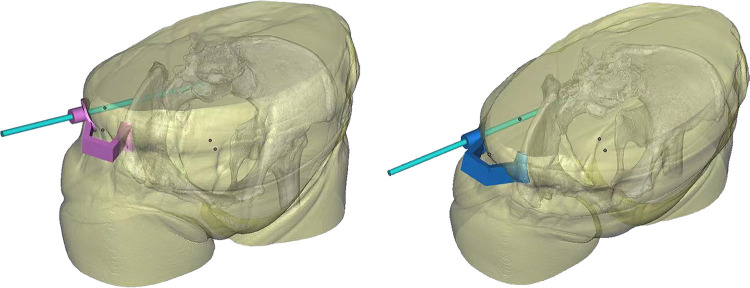
Design of the guide template. (**A**) Example of preoperative planning for S1 screw placement. (**B**) Example of preoperative planning for S2 screw placement.

**Figure 3:**
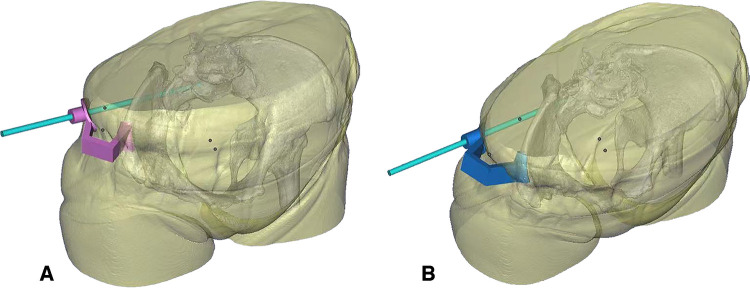
3D printing model of this case. (**A**) The anchoring surface is used to fix the template next to the anterior superior iliac spine. (**B**) The bridging rod is a connection between the anchoring surface and the positioning hole outside the body. The positioning hole is used for percutaneous insertion of the guidewire.

CT scanning of pelvis was preoperatively performed to obtain the three-dimensional reconstruction data. Then, the findings were used for modelling *via* computer-aided design (CAD) and computer-aided manufacture (CAM) technology. The duplicate of the pelvis and the personalized guide template were manufactured with 3D printing technology. Alistone C3850, a 3D printer produced in Guiyang, China, was committed to model manufacturing with a printing accuracy of 0.05–0.4 mm and a printing speed of 20–300 mm/s. The duration of printing this guide template was approximately 42 h. It was intended to navigate the guidewires into the safe zone between the neuroforamen and the cortex of the sacrum accurately (Figure 2). The guide template is composed of three parts (Figure 3): anchoring surface, bridging rod, and positioning hole. The anchoring surface is used to fix the template next to the anterior superior iliac spine, and the bridging rod is a connection between the anchoring surface and the positioning hole outside the body. The positioning hole is used for percutaneous insertion of the guidewire.

The Siemens SOMATOM Conficence® RT Pro was used for intraoperative scanning. The device, a standard 64-slice dual-energy photon large-aperture CT, with the Direct Density™ technique, greatly broadens the selection of scanning voltage, maintains the accuracy of dose measurement, and obtains more accurate image quality.

### Surgery process

**Figure 4:**
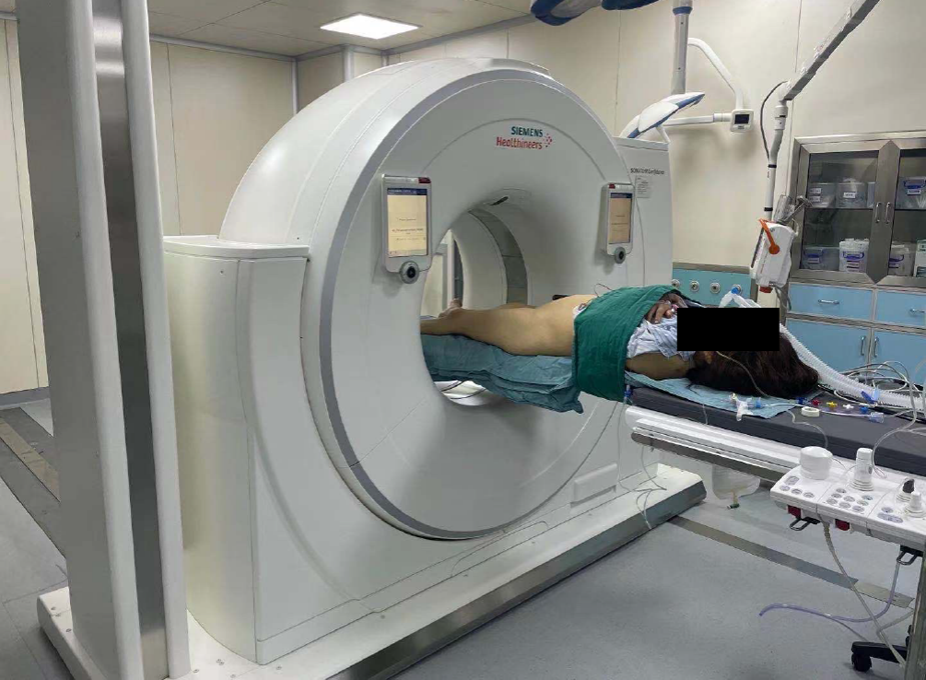
Standard posture of the patients with sacroiliac screw fixation assisted by intraoperative CT.

**Figure 5:**
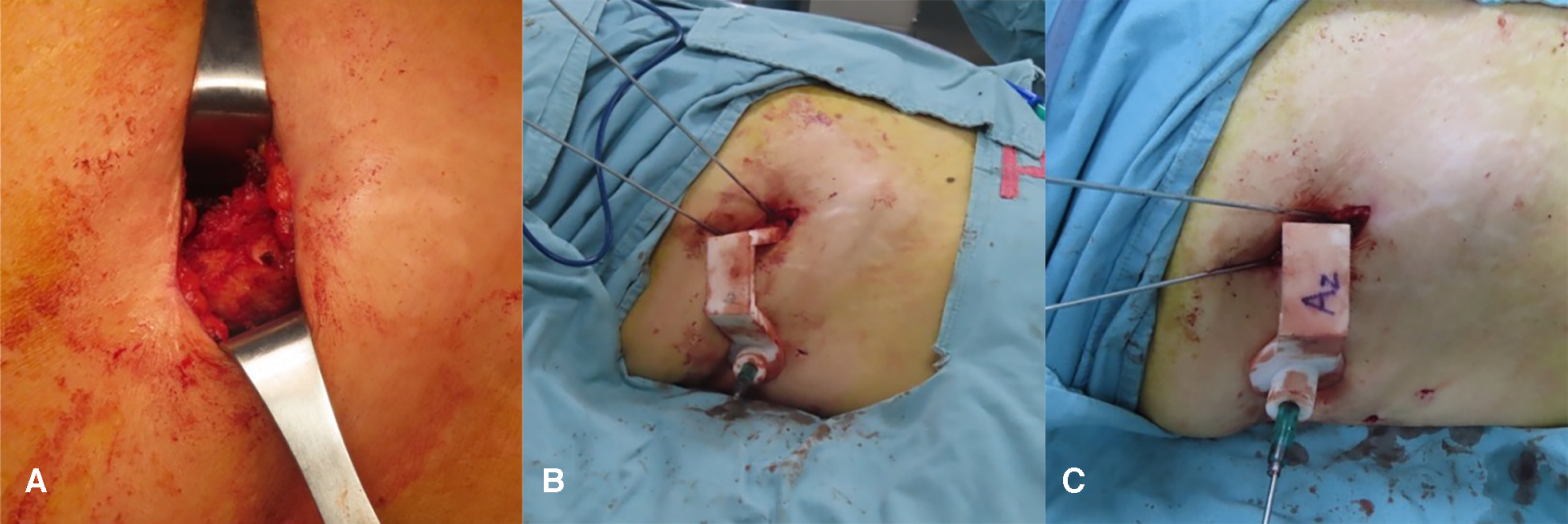
The processes in inserting guidewires with 3D printing guide template. (**A**) Make an incision on the body surface next to the anterior superior iliac spine. (**B,C**) Anchor the S1 and S2 screw guide templates at the iliac spine respectively and place the guidewires along the positioning hole.

**Figure 6:**
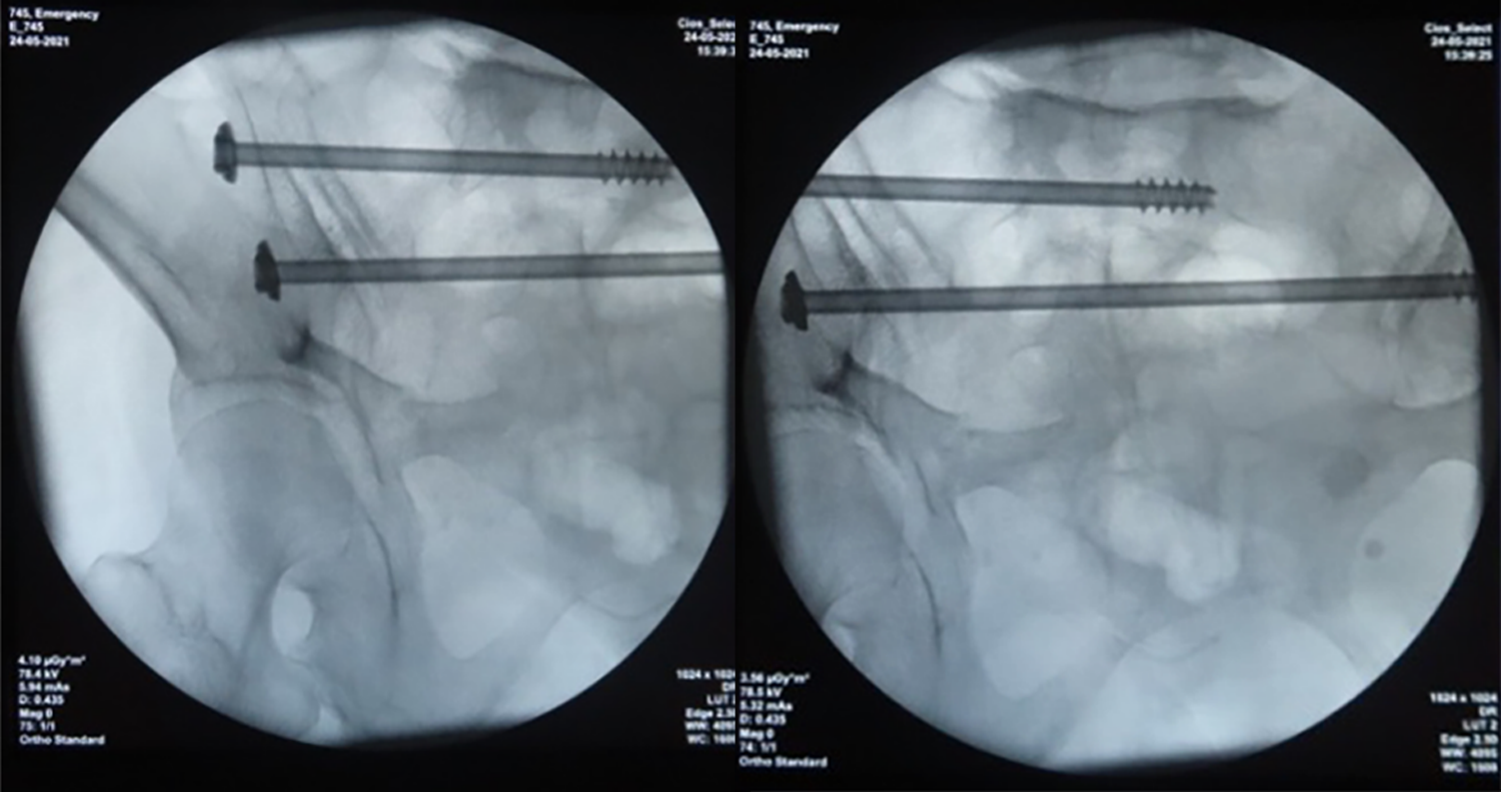
Radiographic imaging with C-arm.

**Figure 7:**
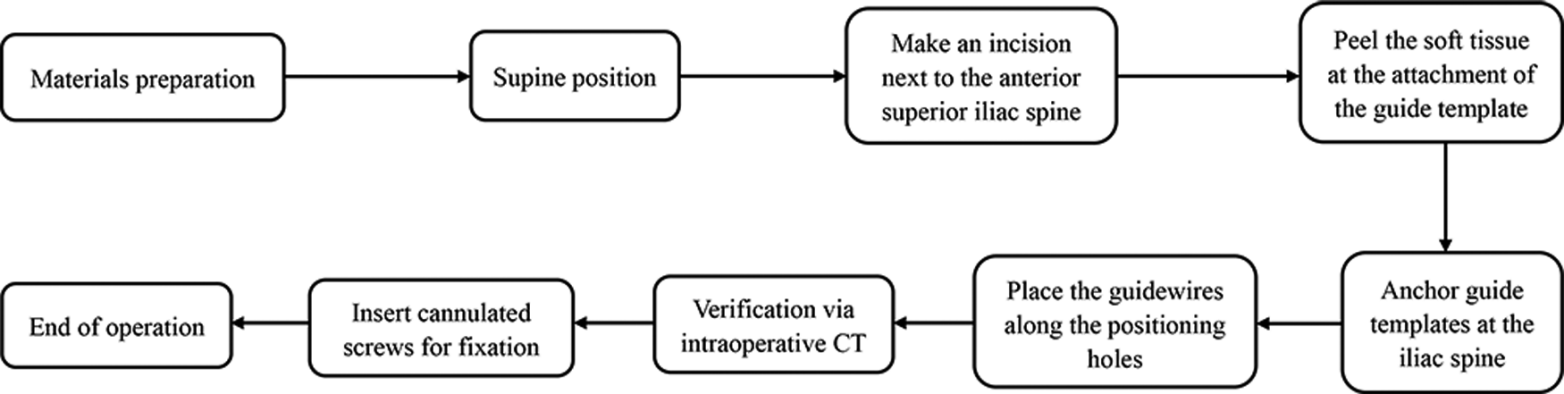
The operation workflow.

**Figure 8:**
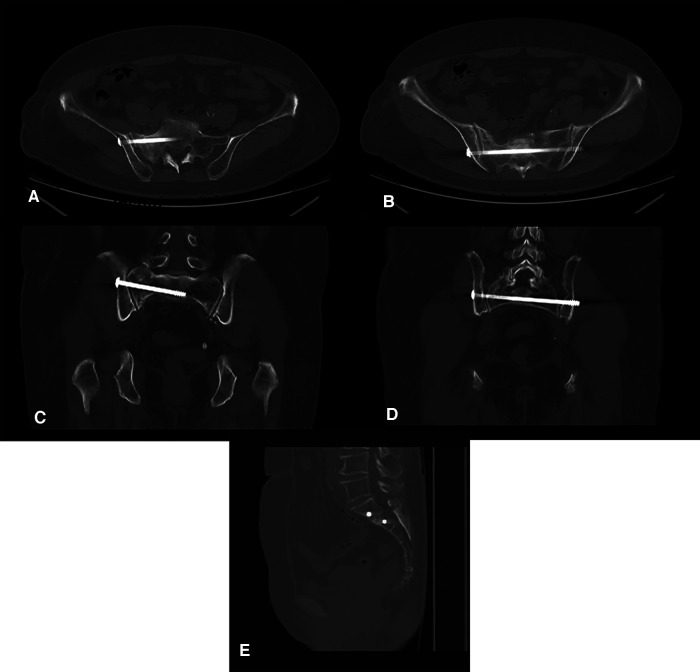
Imaging verification with rail-bound CT during operation. (**A,B**) Horizontal view. (**C,D**) Coronal view. (**E**) Sagittal view.

The patient positioned supine on a radiolucent table in an operating room integrated with mobile intraoperative CT scanner, and the standard posture was shown in the Figure 4. Make an incision on the body surface next to the anterior superior iliac spine, anchor the S1 and S2 screw guide templates at the iliac spine respectively, and place the guidewires along the positioning hole (Figure 5). After the placement was verified with rail-bound CT, cannulated screws were inserted for fixation (Figure 6). The operation workflow was shown in Figure 7. If the position of the guidewires was not good under intraoperative CT verification, the differences of the angle and distance would been measured *via* computing technology and would make timely and accurate adjustment during operation. The screws were accurately placed in the safe zone as shown in the Figure 8. In addition, during the operation, it is necessary to peel the soft tissue at the attachment of the guide template and completely cut the fascia with obvious tension, so as not to affect the accuracy of the guide template.

### Data analysis

The blood loss (ml) calculated by weighing the blood-soaked gauze. The amount of blood loss (g) = the weight of gauze after wiping all blood loss—the weight of dry gauze (1 g = 1 ml). The intraoperative blood loss was 23.03 ml, and the duration of operation was 62 min. The volume computed tomography dose index (CTDlvol) refers to the average dose of slice in the whole spiral scanning volume. Dose length product (DLP) is used to evaluate the total exposure dose during CT scanning. DLP (mGy·cm) = CTDlvol (mGy) * L (cm), L is the length of scanning. mSv = mGy * *k*, *k* is a constant and the value of *k* for the pelvis is 0.015. The exposure dose during CT scanning was 7.025 mSv. The assessment of screws position was excellent based on the criteria of Gras et al. (20). No signs of iatrogenic vascular and nerve injury and other complications were found after operation.

All authors declare that all methods were carried out in accordance with relevant guidelines and regulations and all experimental protocols were approved by Hunan Provincial People's Hospital ethics committee. Written informed consent was obtained from the subject and legal guardian for the publication of any potentially identifiable images or data included in this article.

## Discussion

The structure of pelvic ring is crucial for weight-bearing and mobilization, which is so firm that the disruption of pelvic ring is most frequently occurred in high-energy events (21). The pelvic ring injury is complex since it is commonly accomplished with multiple injuries and may process to dramatic and uncontrolled multiple organ dysfunction syndrome (22). The conventional fixation methods are subjected to more blood loss, longer procedure time, and higher risk of infection, which hinders the recovery and may lead to secondary injury. The minimally invasive method, sacroiliac screws fixation, is gradually substitute for conventional methods in the treatment of posterior pelvic ring injuries (23). However, the method is challenging, necessitates precise placement of implants to avoid serious complications (24). Only a minimal deviation can cause an accurate penetration of the S1 foramina or the presacral cortex when fixed with the sacroiliac screws (25).

3D printing personalized guide template, a rising and precise minimally invasive technology, is gradually being introduced to assist the surgery (17, 18). Personalized guide templates can be customized according to specific injuries. It is expected to navigate the insertion of sacroiliac screws, diminish deviation, and avoid complications. However, at present, there is no study on the application of 3D printing guide template in the sacroiliac screw fixation. We tried to use 3D printing guide template to navigate sacroiliac screw insertion to evaluate the possibility of applying this technique in patients with posterior pelvic ring injury. We report for the first time that 3D printing personalized guide template is used for treatment of posterior pelvic ring injuries with sacroiliac screws under the intraoperative rail-bound CT verification and assistance. In this case, 3D printing personalized guidance template was successfully assist sacroiliac screws in the treatment of posterior pelvic ring injury.

The volume of bleeding in this patient was 23.03 ml, which was better than our usual data. Percutaneous sacroiliac screws fixation usually requires only a smaller incision than plate fixation, allowing a minimal blood loss (26). With the assistance of 3D printing guide template, the insertion point of guidewires can be directly located to reduce the chances of extending the incision. The vascular damage also can be prevented, which may lead to catastrophic bleeding and hemorrhagic shock. In term of the operative duration, previous studies have shown that the application of a new assistive technique in the navigation of internal fixation often leads to a potential increase in the length of operation due to the additional preparation and execution (27, 28). However, in this case, we found that the time of sacroiliac screw implantation can be shortened with the aid of guide template. On the one hand, redundant surgical steps in the surgical process can be avoided. On the other hand, the times of repeatedly adjusting the fluoroscopy to evaluate the screw direction can be reduced. Even the hidden gaining of averting unexpected anesthesia and surgery to correct screw dislocation have not been considered. If the technique is skilled enough, the surgical time would be further shortened. After all, this is only our first attempt.

Perhaps another issue that needs more attention is radiation dosage. It is well known that radiation exposure in medicine is by no means benign. It can cause diseases and injuries, whether to patients or surgeons, accounting for approximately 0.6%–3% of the cancer cases (29). It is reported that the radiation exposure of minimally invasive techniques is higher than that of open surgery (30). In this case, the intraoperative CT was used to verify the effect of the guide template and ensure the safety of patient during operation. And the intraoperative CT showed that the orientation of the guide template was almost accurate. Traditional procedures of sacroiliac screws require more fluoroscopy and time, in contrast there are less times with the assistance of guide template. Furthermore, the surgeons are closest to the radiation source during the conventional procedures of sacroiliac screws, whereas for guide template, they are not. Potential damage from radiation exposure can also be minimized by guide template.

As for complications, the frequency of intraoperative fluoroscopy and the possibility of prolonging the incision due to poor positioning can be reduced through the navigation of the guide template. Secondary traumatic bleeding caused by surgery was also reduced. There were no screws loosening or failure postoperatively. Intraoperative CT could provide a better image quality in the critical anatomical regions of the pelvis. And according to Gras et al. (20), the quality of screws position in this case is excellent. Revision surgery is usually occurs in screws misplacement under conventional approach. An obviously increased risk of adjacent nerves and blood vessels would occur when the displacement was larger than 10 mm (31). Moreover, there was no iatrogenic vascular nerve injury in this case. Up to 8% of patients receiving sacroiliac screw fixation have nerve-related injuries and symptoms (32). When using sacroiliac screw fixation to treat pelvic posterior ring injury, surgeons must be more careful to avoid iatrogenic injury (33–35). In sum, it suggested that the use of 3D printing guide template technology can facilitate intraoperative navigation and reduce the possibility of screws placement into the sacral foramens and the occurrence of iatrogenic injury.

Finally, as far as the economic cost is concerned, the current cost of 3D printer system is between 50,000 and 100,000 RMB. The treatment requires an additional payment for 3D printing, which is about 1,000 to 2,000 RMB, depending on the size of the printing model and the number of customized guide templates. Although a part of the economic burden is increased, the expected benefits must be considered. The estimated cost of revision surgery is approximately 30,000 to 40,000 RMB. This potential risk expenditure is 15–40 times of the cost of 3D printing technology. The time in the operation and the cost of revision surgery for symptomatic screw misplacement can be saved *via* 3D printing guide template, which can be regarded as the return on investment of 3D printing technology purchase. The use of 3D printer by multiple disciplines further increases the return on investment. Finally, the use of such a device is conducive to improve patient safety, which is what we are pursuing. Taking all these factors into account, the benefits of 3D printing guide templates for sacroiliac screws far outweigh the disadvantages from an economic perspective.

There are also several limitations in current study. Firstly, these are just conjectures obtained from a common case, in nature, which was prone to random variations and uncontrolled bias. Secondly, only one device can be relied on, and more data originated from other similar systems is required. Thirdly, there is a limiting factor in the implementation and development of such an intraoperative CT and 3D printer, which requires high investment, so that it is relatively rare in general hospitals. Fourth, there is no follow-up data for this case currently. The research subject will continue be followed up. If feasible, a multicenter study will be performed, and more cases will be included in the future. Fifth, this treatment may only apply to pelvic fractures within type B of Tile classification. Finally, obesity may pose some risk of bias between the 3D template and actual condition when performing percutaneous sacroiliac screw, affecting the accuracy of the guide template. In view of the above problems, trials with larger samples and higher quality can be carried out in the future to better control confounding variables and improve the reliability of findings. Furthermore, separate radiation protection guidelines should be proposed and followed to better protect patients.

## Conclusion

To summarize, we believe that a reduction of surgical duration and blood loss with intraoperative assistance of 3D printing personalized guide template could be obtained. Less misposition and functional impairment could be achieved, and a more satisfactory curative effect could be developed. Therefore, sacroiliac screws fixation with the assistance of 3D printing personalized guide template may be a promising method to treat posterior pelvic ring injuries. Of course, in view of the sample size, more cases, more practice, and more frequent use are needed to further study the existence of this difference.

## Data Availability

The original contributions presented in the study are included in the article/Supplementary Material, further inquiries can be directed to the corresponding author.
